# Improved tactile speech perception and noise robustness using audio-to-tactile sensory substitution with amplitude envelope expansion

**DOI:** 10.1038/s41598-024-65510-6

**Published:** 2024-07-01

**Authors:** Mark D. Fletcher, Esma Akis, Carl A. Verschuur, Samuel W. Perry

**Affiliations:** 1 University of Southampton Auditory Implant Service, University of Southampton, University Road, Southampton, SO17 1BJ UK; 2 Institute of Sound and Vibration Research, University of Southampton, University Road, Southampton, SO17 1BJ UK

**Keywords:** Auditory system, Sensory processing, Somatosensory system, Biomedical engineering, Translational research

## Abstract

Recent advances in haptic technology could allow haptic hearing aids, which convert audio to tactile stimulation, to become viable for supporting people with hearing loss. A tactile vocoder strategy for audio-to-tactile conversion, which exploits these advances, has recently shown significant promise. In this strategy, the amplitude envelope is extracted from several audio frequency bands and used to modulate the amplitude of a set of vibro-tactile tones. The vocoder strategy allows good consonant discrimination, but vowel discrimination is poor and the strategy is susceptible to background noise. In the current study, we assessed whether multi-band amplitude envelope expansion can effectively enhance critical vowel features, such as formants, and improve speech extraction from noise. In 32 participants with normal touch perception, tactile-only phoneme discrimination with and without envelope expansion was assessed both in quiet and in background noise. Envelope expansion improved performance in quiet by 10.3% for vowels and by 5.9% for consonants. In noise, envelope expansion improved overall phoneme discrimination by 9.6%, with no difference in benefit between consonants and vowels. The tactile vocoder with envelope expansion can be deployed in real-time on a compact device and could substantially improve clinical outcomes for a new generation of haptic hearing aids.

## Introduction

Large recent advances in haptic technology mean that haptic aids for hearing, which provide sound information through tactile stimulation on the skin, could now play an important role in supporting those with severe or profound hearing loss^1,2^. In the past, these haptic hearing aids (also called “tactile aids”) were used clinically to support speech perception in people with hearing loss (e.g.,^3^). While they were shown to be effective for tactile-only word recognition^4^ and for supporting lip reading^3,5, 6^, by the mid-1990s large improvements in the effectiveness of cochlear implants (CIs) and limitations in haptic technology meant that haptic hearing aids were rarely used clinically^2^. Since then, the key technologies for haptic hearing aids, such as compact actuators, batteries, and microprocessors, have progressed hugely. If a new generation of haptic devices can be shown to effectively transfer speech information, they could dramatically improve outcomes for the many millions of people with a profound hearing loss who are unwilling to use CIs or who are unable to access them because they are expensive and invasive^2,7^. Additionally, they may assist individuals who have a severe hearing loss but cannot use hearing aids for medical reasons, such as allergic responses to ear mould materials or external ear disease. If haptic hearing aids can additionally be shown to be robust to background noise, then they could also be an important supplement to CI technology, whose users often struggle to understand speech in background noise^8,9^.

Recent developments in wide-band haptic technology mean that it is now possible for a compact wearable device to deliver haptic stimulation across a relatively wide frequency range. This has made frequency-to-frequency audio-to-tactile conversion viable for haptic aids. Several recent studies have exploited this using a tactile vocoder approach^8–15^. In this approach, audio is filtered into frequency bands and the amplitude envelope for each band is extracted. Each of these envelopes is used to modulate the amplitude of one of several vibro-tactile tones. These tones are delivered through vibro-tactile stimulation at a single site, most commonly on the wrist (as it is a suitable site for a real-world wearable device^1^). The tactile vocoder approach allows the frequency range of speech to be converted to the frequency range where the tactile system is most sensitive to vibration^16,17^. This approach has been shown to effectively transfer phonemic and other speech information for haptic stimulation alone^12,14, 15^ and to improve speech recognition in background noise^8,9, 11^ and sound localisation^10,13, 18, 19^ when haptic stimulation is used to augment the electrical CI signal (“electro-haptic stimulation”^8^).

The latest tactile vocoder method effectively transfers important consonant information, such as the presence or absence of voicing and differences in manner or place of articulation, but is poor at transmitting vowel information^12^. Previously, multi-band dynamic-range expansion has been used to enhance key phonemic features, such as formants, which are critical to vowel perception^20^. Expansion works in the opposite way to compression, in that that it expands the dynamic range of the signal rather than compressing it. Expansion is often performed only above a certain threshold, so that only more intense portions of the signal are exaggerated. When applied separately across different frequency bands, this can result in key high-intensity and spectrally focused speech features, such as formants, being emphasised. The first aim of the current study was to test whether multi-band amplitude envelope expansion with the tactile vocoder can improve tactile phoneme discrimination in quiet.

Envelope expansion was expected to improve performance most for vowels, predominantly through enhanced formant representation. A smaller, but still significant, improvement with envelope expansion was expected for consonants, because of emphasised mid-to-high frequency spectral tilt, formants, and amplitude envelope cues. Mid-to-high frequency spectral tilt is important to obstruent consonant perception, formant representation is important for sonorant consonant place and manner of articulation perception, and envelope cues are important for conveying manner of articulation.

The second aim of the current study was to test whether envelope expansion enhances phoneme discrimination in background noise. To prevent distortion, envelope expansion is often followed by a reduction in gain^8,11^. This means that, in addition to exaggerating more intense portions of the signal, less intense portions are suppressed. In scenarios where the target speech is louder than the background noise, there is evidence that this can improve extraction of speech from background noise with the tactile vocoder^8,9, 11^. In the current study, the noise was set to 5 dB below the level of the speech, which is an SNR where the tactile vocoder without envelope expansion is known to breakdown^8,11^ and is worse than is found in many common challenging real-world environments for hearing-impaired listeners^21^. It is also an SNR at which CI users struggle substantially^8,9^, and so if the envelope expansion is found to be effective under these conditions, it would indicate that it may be a useful method for electro-haptic stimulation.

Envelope expansion was expected to improve the representation of speech in noise by enhancing the higher intensity speech and suppressing the lower-intensity background noise. Note that it is therefore only expected to be effective when speech is more intense than the background noise (see^8,11^). This enhancement would be expected to apply for both consonants and vowels for phonemes in noise. Envelope expansion was also expected to enhance onset cues and other fast amplitude envelope changes and thereby particularly aid performance in noise for consonants differing by both manner and place of articulation, which heavily rely on these cues^22^.

## Results

**Figure 1:**
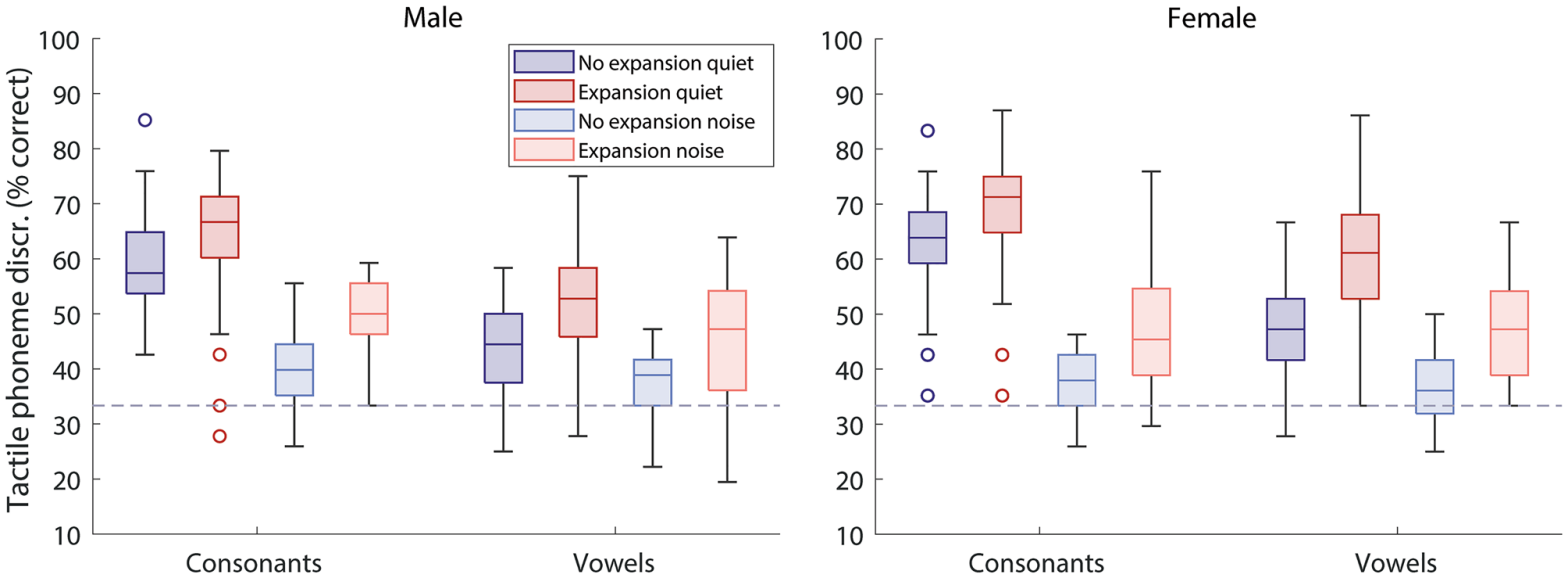
Box plots showing the percentage of phoneme pairs discriminated with (red/orange shading) and without (purple/blue shading) envelope expansion. Consonants and vowels are shown separately for the male (left panel) and female (right panel) talkers, both in quiet and in background noise. The horizontal line inside the boxes shows the median and the top and bottom edges of the boxes show the upper (0.75) and lower (0.25) quartiles. Unfilled circles show outliers (more than 1.5 times the interquartile range). The whiskers connect the upper and lower quartiles to the maximum and minimum non-outlier values. The dashed grey line shows chance performance.

Figure 1 shows the percentage of consonants and vowels correctly discriminated in each experimental condition for the 32 participants who took part in the current study. Across all phonemes in quiet, the envelope expansion improved performance by 7.6% on average (ranging from -4.4 to 17.8%; standard deviation (SD) of 5.5%; *F*(1,31) = 66.1, *p* < 0.001; partial eta squared (η^2^) = 0.681). A significantly larger improvement in quiet was observed for vowels than for consonants (interaction between phoneme type and presence or absence of envelope expansion: *F*(1,31) = 7.3, *p* = 0.011; η^2^ = 0.190). For consonants in quiet, the mean improvement with envelope expansion was 5.9% (ranging from -7.4 to 17.6%; SD of 6.6%) and, for vowels, was 10.3% (ranging from -2.8 to 27.8%; SD of 7.9%). Post-hoc testing showed that, while envelope expansion had a different effect on performance for vowels and consonants, it nonetheless provided a significant benefit with both phoneme types (consonants: *t*(31) = 5.0, *p* < 0.001, Cohen’s *d* = 0.58; vowels: (*t*(31) = 7.4, *p* < 0.001, *d* = 1.14).

Envelope expansion was found to have larger overall benefit for the female talker in quiet than for the male talker (interaction between talker and presence or absence of envelope expansion: *F*(1,31) = 7.0, *p* = 0.013; η^2^ = 0.183). For the female talker, the mean improvement with envelope expansion was 9.6% (ranging from -12.2 to 25.6%; SD of 7.7%) and, for the male, was 5.7% (ranging from -12.2 to 16.7%; SD of 6.9%). The larger benefit of envelope expansion in quiet for vowels was found only for the female talker (significant three-way interaction between envelope expansion, talker, and phoneme type: *F*(1,31) = 4.2, *p* = 0.048; η^2^ = 0.120). For the male talker, envelope expansion improved performance by 6.5% (SD of 9.5%) for vowels and 5.1% (SD of 9.5%) for consonants. For the female talker, envelope expansion improved performance by 14.1% for vowels (SD of 11.5%) and 6.6% for consonants (SD of 7.7%).

In background noise, envelope expansion improved performance by 9.6% on average across all phonemes (ranging from -1.1 to 24.4%; SD of 6.4%; *F*(1,31) = 65.1, *p* < 0.001; η^2^ = 0.677). No difference in the size of this benefit was found across the two talkers (*F*(1,31) = 0.8, *p* = 0.378) or phoneme types (*F*(1,31) = 0.3, *p* = 0.566). Post-hoc testing confirmed the effect of envelope expansion in noise was significant both for consonants (*t*(31) = 8.4, *p* < 0.001; *d* = 1.73) and vowels (*t*(31) = 5.4, *p* < 0.001; *d* = 1.15).

**Figure 2:**
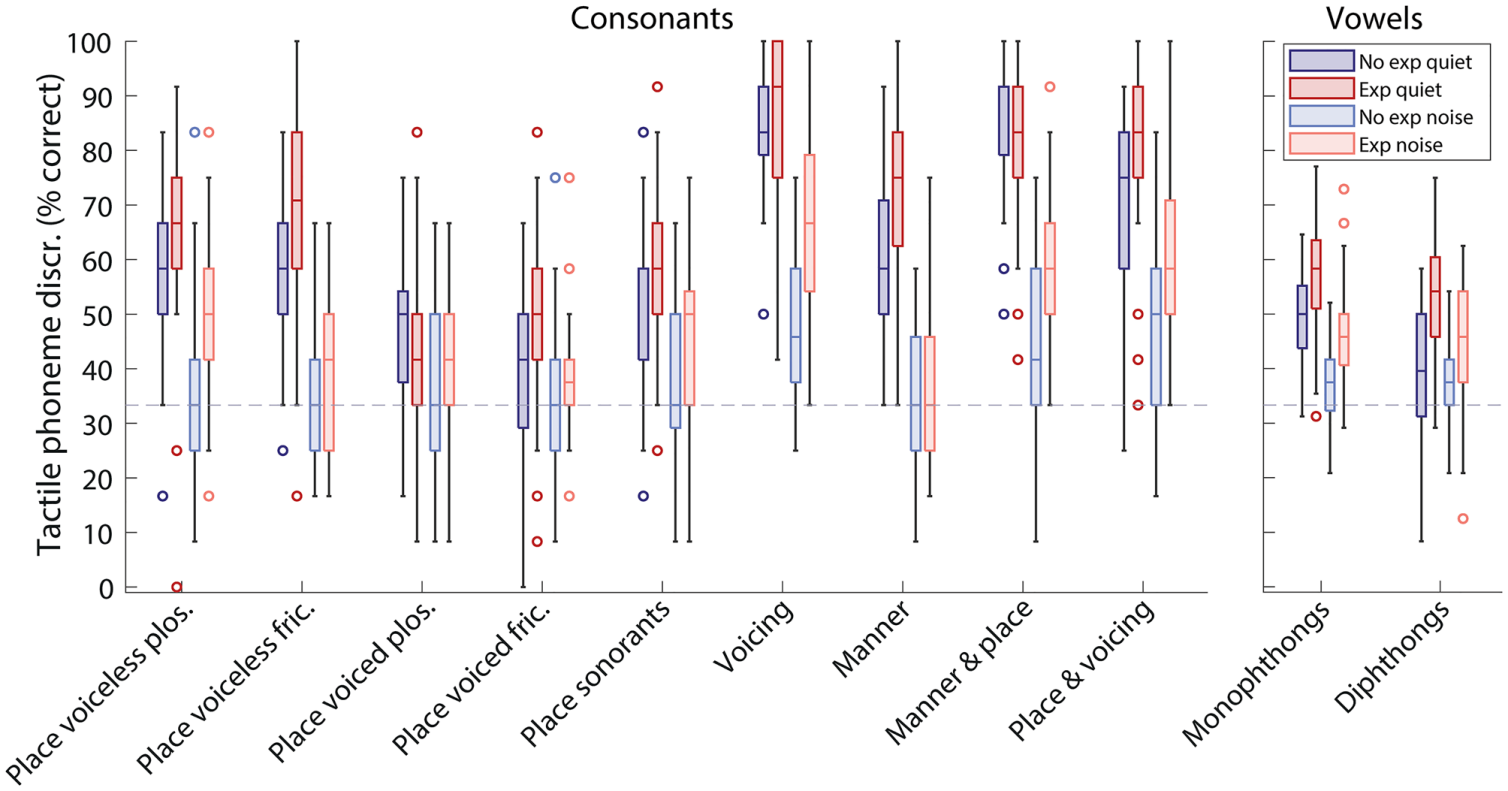
Box plots (as in Fig. 1) showing the percentage of phonemes discriminated for each condition, grouped by phoneme contrast type. Chance performance is marked with a dashed grey line.

Figure 2 shows performance for different phoneme contrast types (see “Methods”). Post-hoc analyses (corrected for multiple comparisons) showed no significant improvements with envelope expansion for any consonant contrasts in quiet. However, improvement with envelope expansion was close to significance for pairs differing by both place of articulation and voicing (mean difference: 10.2%; SD: 18.1%; *t*(31) = 3.2, *p* = 0.051) and for voiced fricative pairs differing by place of articulation (mean difference: 11.7%; SD: 22.1%; *t*(31) = 3.0, *p* = 0.074). For vowels in quiet, envelope expansion improved performance for both monophthongs, with a mean improvement of 8% (SD: 8.3%; *t*(31) = 5.5, *p* < 0.001; *d* = 0.77), and diphthongs, with a mean improvement of 14.8% (SD: 14.1%; *t*(31) = 6.0, *p* < 0.001; *d* = 1.29).

Post-hoc analyses also revealed significant improvements with envelope expansion for some consonant contrast types in background noise. Envelope expansion improved performance by, on average, 19.3% for pairs differing by whether or not they were voiced (SD: 21.2%; *t*(31) = 5.1, *p* < 0.001; *d* = 0.45), 17.2% for pairs differing by both manner and place of articulation (SD: 20.7%; *t*(31) = 4.7, *p* < 0.001; *d* = 0.29), and 13.3% for pairs differing by both place of articulation and whether they were voiced (SD: 23.2%; *t*(31) = 3.2, *p* = 0.048; *d* = 0.57). Improvement in performance with envelope expansion was close to significance for voiceless plosives differing by place of articulation (mean difference: 14.8%; SD: 26.3%; *t*(31) = 3.2, *p* = 0.051). For vowels in background noise, envelope expansion improved performance only for the monophthongs, with a mean improvement of 10.2% (SD: 11.3%; *t*(31) = 5.1, *p* =  < 0.001; *d* = 1.15).

Finally, exploratory analyses assessed whether there was a correlation between either age or detection thresholds for a 125 Hz vibro-tactile tone (measured during screening) and phoneme discrimination in quiet, either with or without envelope expansion. No evidence of a correlation between either age or detection thresholds and phoneme discrimination was found.

## Discussion

The current study showed clear benefit of multi-band amplitude envelope expansion to tactile phoneme discrimination both in quiet and in background noise. As anticipated, in quiet a larger benefit of envelope expansion was observed for vowels than for consonants and in noise benefit was similar for vowels and consonants. These results suggest that envelope expansion could be a highly effective audio-to-tactile signal-processing technique for both enhancing important phonetic cues in quiet and extracting speech from background noise.

**Figure 3:**
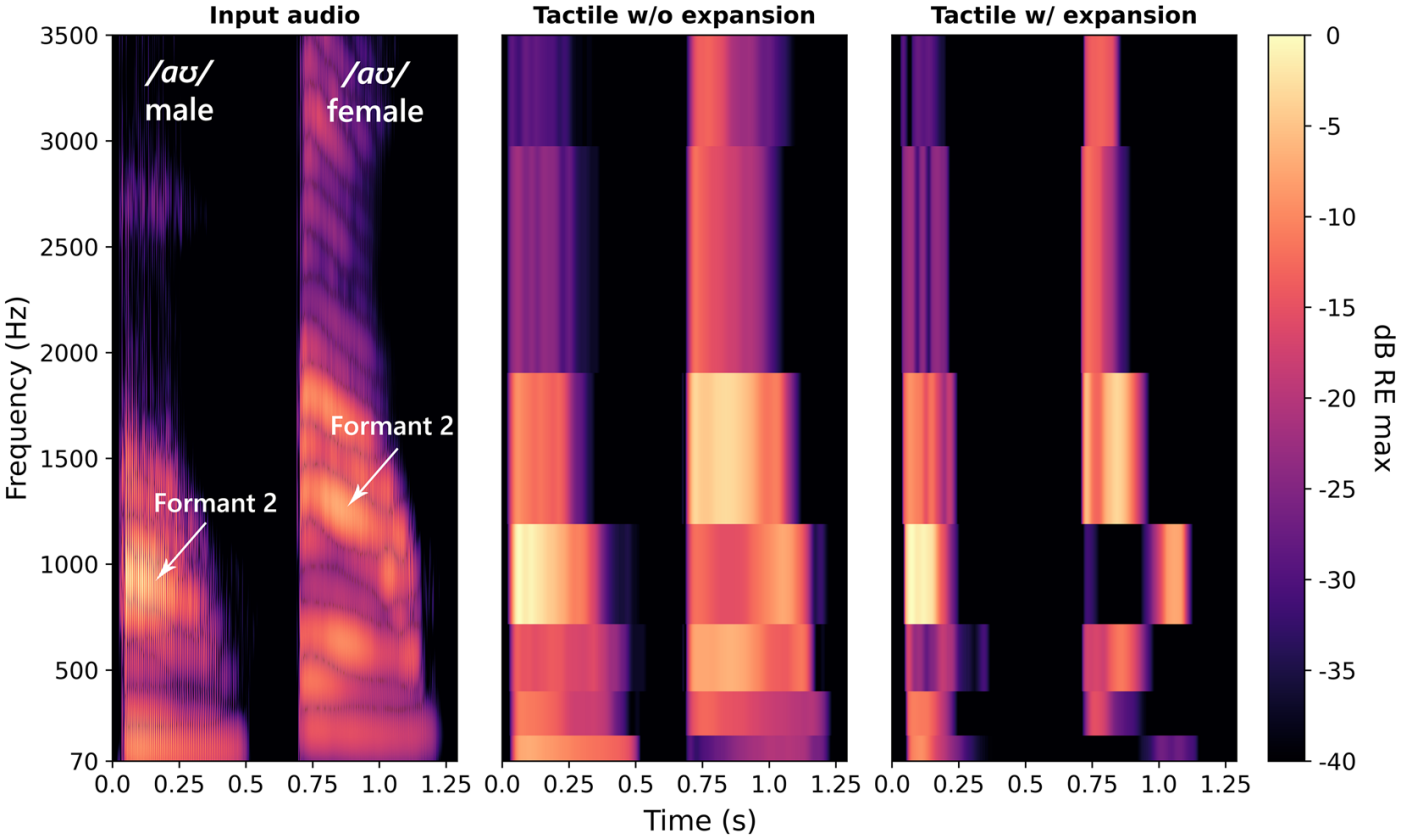
Spectrograms showing the input audio (left panel), the tactile envelopes without expansion (central panel) and the tactile envelopes with expansion (right panel). The vowel /aʊ/is shown, with the first token spoken by the male talker and second token spoken by the female talker. The second formant for each token is marked on the input audio. For clarity, the frequency range plotted is focused on the formants. The audio spectrogram sample rate was 22.05 kHz, and a 8-ms Hann window with a hop size of 1 sample was used. Windows were zero-padded to 8192 samples. The sample rate for the tactile spectrograms was 16 kHz, with no windowing applied. For the input audio, intensity is shown in decibels relative to the maximum magnitude of the short-time Fourier transform. For the tactile envelopes, intensity is shown in decibels relative to the maximum envelope amplitude. The spectrograms were generated using the Librosa Python library (version 0.10.0).

In quiet, envelope expansion improved vowel discrimination more for the female talker than for the male talker. For the female talker, the formants may have been better resolved because of better alignment of formant frequencies with the tactile vocoder audio frequency band limits, which could have facilitated greater enhancement with envelope expansion. The larger formant frequency glides for the female talker might also have allowed formant changes to be better resolved with the limited spectral resolution available. Figure 3 shows an example of greater formant enhancement with envelope expansion for the female talker. The audio and tactile signals for the vowel /aʊ/ (as in “m**ou**th”) are shown for the male and female talker. For the male talker, the downward frequency glide for the second formant is small and is not portrayed in the tactile envelopes, either with or without envelope expansion. In contrast, the second formant frequency glide for the female talker is represented in non-expanded tactile envelopes and is enhanced after envelope expansion is applied, with a substantial reduction in the energy in frequency bands adjacent to the formant.

For pairs differing by whether they were voiced, there was also no evidence of a benefit of envelope expansion in quiet, but there was a clear benefit in noise. The absence of benefit in quiet was likely due to the fact that the voicing bar (~ 150–200 Hz) was already well represented without envelope expansion (as shown previously^12,14^), meaning that any enhancement of this cue could not impact performance. In noise, however, masking of the voicing bar would have made it less salient without envelope expansion applied (as indicated by the large reduction in performance in noise). Envelope expansion may have reduced masking by enhancing the representation of the voicing bar and suppressing less intense signals in other frequency bands.

Performance for consonant pairs differing by manner and place of articulation was improved in noise but not in quiet. This improved performance was anticipated, as envelope expansion improves the salience of fast onset amplitude envelope changes, which are crucial to these contrasts^22^. Like for voicing cues, these cues may have been sufficiently salient in quiet even without envelope expansion (as indicated by the high performance in this and previous work^12,14^), but have been made more salient by envelope expansion in noise.

The current study has a number of limitations. Firstly, the participants were different to those who are expected to use haptic hearing aids. All participants were under 40 years of age, but many future haptic hearing aid users are likely to be older than this. Like in previous work^12,14^, we found no evidence of a correlation between phoneme discrimination and either age (which spanned 20 years in the current study) or tactile sensitivity. In previous work on haptic enhancement of speech-in-noise performance in CI users, there has also been no evidence of a relationship between age or tactile sensitivity and the amount of haptic enhancement^8,9,11,12^. In addition, no relationship has been observed between age and either tactile intensity discrimination^18,23^ or temporal gap detection for vibro-tactile tones^24^. The results of these studies suggest that the current findings would generalise to older users. However, absolute vibro-tactile detection sensitivity^18,25^ and frequency discrimination^26^ are both known to decline with age. This could lead to reduced overall tactile speech performance for older haptic device users but might also allow greater benefit of envelope expansion as it can enhance the frequency and amplitude representation of important phonemic cues (as shown in Fig. 3).

As well as not matching the age range of anticipated haptic hearing aid users, the participant group in the current study did not include individuals with hearing impairment. Across several studies, no differences in tactile speech performance between normal-hearing and hearing-impaired individuals has been found^8,11,12,27,28^. For example, studies of electro-haptic stimulation have found similar benefit to speech-in-noise performance for CI users and for normal-hearing individuals listening to CI simulated audio^8,11^. However, future work is required to authoritatively establish whether tactile speech perception differs across target user groups and those without hearing impairment.

A further limitation of the current study is that the phoneme discrimination task used does not assess the participant’s ability to detect temporal boundaries of phonemes, syllables, or words in running speech (segmentation). Discrimination was preferred to identification, because an identification task would require an impractically long training regime (potentially lasting several months^4^) to achieve good generalisability across a wide stimulus set. The improved discriminability of phonemes with envelope expansion that was found in the current study would be expected to facilitate better separation of phonemes in running speech^29^ (although this relationship between phoneme discrimination and speech segmentation has not yet been established for tactile speech perception). Envelope expansion might also be expected to improve segmentation by enhancing key landmarks such as phoneme and syllable onsets. The use of isolated phonemes in the task meant that perception of phoneme transitions, other than those in diphthongs, was not assessed. The large improvement in performance for diphthongs with envelope expansion might indicate that perception of these transitions will be enhanced. However, it will be important for future work to assess the impact of envelope expansion on tactile perception of running speech.

The phoneme discrimination task was used to determine the limits of discriminability for phonemically relevant acoustic information. Studies of categorical perception of speech have shown that discrimination of acoustic contrasts that signal different phoneme identities is a necessary precondition for successful speech recognition^30^. However, envelope expansion may have improved discrimination by enhancing cues that are not useful for phoneme identification. The non-uniform and explicable pattern of results across different phoneme contrast types suggests that performance did not improve because of a general increase in variability in the tactile representation. For example, in quiet there was no evidence of a benefit of envelope expansion for voiced plosives that differed by place of articulation or phonemes that differed by the presence of voicing or by manner and place of articulation. In contrast, there were clear benefits in quiet for monophthong and diphthong vowel contrasts, which were anticipated. It is also important to note that, when there was background noise, the discrimination task precluded the use of cues from the noise by using a different noise token in each observation interval. For conditions both in quiet and in noise, the use of absolute intensity cues was also precluded by roving the intensity of the stimuli across intervals. The improvement in phoneme discrimination with envelope expansion would therefore be expected to translate to improved phoneme identification, although the relationship between tactile phoneme discrimination and identification is yet to be firmly established.

It is possible that, when enhancing high-intensity phonemic cues such as formants, envelope expansion impaired perception of less energetic cues, such as low-intensity frication noise for some fricatives or less-intense higher frequency formants for nasal phonemes. Because of the limited frequency resolution and relatively small dynamic range available through vibro-tactile stimulation (~ 55 dB on the wrist^19^) compared to audio with normal-hearing, high intensity cues that are more important for audio speech perception^31^, such as the first and second formants, were prioritised. Further work is required to establish which speech cues are most effectively perceived through tactile stimulation and whether the cues that are most important when listening to speech are also the most important when feeling speech through tactile stimulation.

When considering ways to improve haptic hearing aids, it is important to understand the limitations of the tactile system. The tactile system has around four times the dynamic range available through electrical CI stimulation and can perceive around double the number of intensity steps^18,19, 23,32–34^. It is also highly sensitive to speech amplitude envelope frequency differences in the range most important for speech perception^35–37^. It is therefore well suited for portraying sound intensity information. This is likely why approaches that rely on amplitude envelope information have been effective for haptic hearing aids. In contrast, temporal precision^38–40^ and frequency discrimination^41,42^ are poorer than for CI listening. Despite having poor frequency discrimination, it has been shown that important speech information can be delivered through frequency differences in tactile stimuli using the tactile vocoder method^12^. It was shown that, for vibro-tactile tones that were sufficiently separated in frequency to be discriminable, more phonemic information could be transferred with eight vibro-tactile tones than with either one or four. Future work should focus on establishing models that can predict tactile speech performance to aid improvement of audio-to-tactile conversion methods. Models already exist that might be valuable for this (e.g.,^43^).

New tactile speech models and strategies for converting audio to tactile stimulation, should carefully consider cross-model influences, as visual information (e.g., lip reading) and auditory information (from residual acoustic hearing or electrical CI stimulation) will be available in many real-world scenarios in which haptic hearing aids would be used. The principal of inverse effectiveness states that multisensory integration is maximal when each sense alone provides relatively low-quality, incomplete information^44,45^. This condition would be well met in many scenarios where tactile stimulation is used to support lip reading and electrical hearing through a CI or acoustic hearing with a profound hearing loss. Another key principle for maximising multisensory integration is temporal coincidence^46^, whereby cross modal stimulation must have a good temporal alignment. Again, this condition would be well met for haptic hearing aids using the tactile vocoder, which has temporally complex stimulation that is highly correlated with lip reading and CI or acoustic stimulation. Furthermore, there is evidence that consistent cross-sensory delays in the arrival of sensory stimulation of many tens of milliseconds are well tolerated^47–49^. This is well within the range of latencies within which the tactile vocoder could be implemented on a compact device.

The benefits of envelope expansion to tactile speech perception should be compared with benefits using other enhancement techniques. For example, one alternative approach to enhance phonemic cues could be dynamic adaption of the focus of audio frequency bands in the tactile vocoder based on the estimated frequency characteristics of the talker of interest. Similar techniques might be deployed to those used for adaptive frequency compression, which has previously been trialled in hearing aids^50^. Other techniques for extracting speech from background noise could also be tested, including more sophisticated recently developed methods that exploit neural networks^15^. When comparing with envelope expansion, it will be important to focus on methods that can be deployed in real time on a compact device, so that they can translate to real-world benefits.

In addition to tactile speech perception, future work should establish the effectiveness of envelope expansion in improving tactile perception for a wider range of sounds, such as music^51^ and environmental sounds^52^. Additionally, future studies should explore the effect of envelope expansion on haptic sound localisation and segregation of spatially separated sounds. Previously, haptic sound-localisation^10,13^ and segregation^9^ strategies have converted the audio signal received at each ear to vibro-tactile stimulation on each wrist. This allows sound intensity differences across the ears (interaural intensity differences), which are a key spatial hearing cue, to be transferred through tactile intensity differences across the wrists^18,19^. When optimising the tactile vocoder approach for haptic sound-localisation or segregation, it is likely to be important to ensure that envelope expansion is linked between the left and right audio channels to avoid distortion of interaural intensity differences^13^.

When building a new generation of haptic hearing aids, it will be important to consider the design priorities that have previously been highlighted for visual sensory substitution devices^53^. For example, devices should be easy to fit, simple to use, and not bulky; they should keep the user’s hands free, and they should not interference with the user’s ability to sense the environment (e.g., through residual hearing or a CI). Ensuring an inclusive design will also be important. This might be achieved by connecting haptic hearing devices to items in the internet of things, such as doorbells, oven alarms, and baby monitors^1^. It might also be achieved by developing haptic hearing aids that can be used for other applications, such as enhancing the experience of virtual reality^54^ or remotely controlling tools^55^.

The current study found that envelope expansion is highly effective at improving tactile phoneme discrimination with the tactile vocoder strategy, both in quiet and in background noise. The tactile vocoder with envelope expansion is computationally lightweight and can be implemented in real time on a compact wearable haptic device. It could substantially improve speech performance for a new generation of haptic hearing aids, both when used in combination with existing hearing-assistive devices, such as CIs, and when used as a low-cost and non-invasive alternative for the many millions of people across the world who currently cannot benefit from CI technology.

## Methods

### Participants

Participant characteristics for the 32 adults who took part in the study are shown in Table 1. The average participant age was 28 years (ranging from 19 to 39 years), with 10 males and 22 females. Participants all had normal touch perception, as assessed by vibro-tactile detection thresholds at the fingertip and a health questionnaire (see "Procedure"). All participants reported having no known hearing impairment. Each participant received a £20 inconvenience allowance for taking part.

**Table 1:** Participant characteristics.

ID	31.5 Hz thresh. (m/s^−2^)	125 Hz thresh. (m/s^−2^)	Wrist temp. (°C)	Wrist height/width (mm)	Wrist circum. (mm)	Dom. hand (L/R)	Age (yrs.)	Sex (M/F)
1	0.02	0.08	30.4	39/58	166	R	36	M
2	0.03	0.07	30.2	36/48	149	L	28	F
3	0.05	0.02	32.7	37/49	169	R	27	F
4	0.04	0.10	29.8	36/46	145	R	25	F
5	0.05	0.03	27.1	44/59	171	R	19	M
6	0.08	0.15	28.3	35/46	144	R	23	F
7	0.03	0.04	30.7	36/48	152	R	20	F
8	0.05	0.07	29.6	30/43	129	R	33	F
9	0.04	0.10	27.5	31/48	139	R	27	F
10	0.04	0.09	28.1	39/48	149	R	30	F
11	0.02	0.05	29.4	31/42	134	R	19	F
12	0.05	0.09	31.1	31/44	142	R	31	F
13	0.05	0.09	30.3	35/54	152	R	29	F
14	0.05	0.06	28	43/58	180	R	27	M
15	0.12	0.08	31.8	40/52	161	R	22	M
16	0.02	0.18	28.1	36/50	158	R	36	F
17	0.04	0.02	30.3	42/65	186	R	25	M
18	0.39	0.23	29.1	42/52	165	R	32	F
19	0.08	0.10	29.5	48/61	188	R	31	M
20	0.06	0.06	32.1	45/60	190	R	31	M
21	0.06	0.14	28.2	32/47	139	R	32	F
22	0.04	0.08	28.1	35/44	136	R	19	F
23	0.16	0.22	29.5	46/63	192	R	31	M
24	0.07	0.15	29.4	35/51	149	R	30	F
25	0.03	0.05	27.5	29/43	132	R	22	F
26	0.01	0.04	32.4	40/55	163	R	26	M
27	0.07	0.10	30.8	37/48	156	R	28	F
28	0.06	0.10	30.3	36/49	142	R	39	F
29	0.05	0.08	32.4	33/43	139	R	19	F
30	0.06	0.18	29.5	42/58	179	R	34	M
31	0.04	0.08	31.2	38/50	147	R	28	F
32	0.05	0.07	31.8	36/51	154	R	28	F
Mean	0.06	0.09	29.9	44/60	156	–	28	–

Vibro-tactile detection thresholds at the index fingertip for 31.5 and 125 Hz sinusoids measured in screening, dorsal wrist temperature during the experiment, wrist dimensions (height, width, and circumference), dominant hand, age, and sex are shown. Detection thresholds were in the normal range if they were below 0.4 m/s^−2^ RMS at 31.5 Hz and 0.7 m/s^−2^ RMS at 125 Hz (see “Procedure”).

### Stimuli

For phoneme discrimination testing, the audio used to generate the tactile stimulus was taken from the EHS Research Group Phoneme Corpus^12^. This contains a British English male talker, with an average fundamental frequency of 145.4 Hz, and a British English female talker, with an average fundamental frequency of 208.2 Hz. For each talker, there are four recordings of each of the 44 UK British English phonemes. A subset of 45 phoneme pairs was used in the phoneme discrimination task (see “Table 2”). Pairs were selected to ensure a wide range of phoneme contrasts, including those where discrimination cues are not available through lip reading or through hearing for those with a substantial high-frequency hearing loss (a common loss profile). Pairs also included common vowel and consonant confusions for CI users^56^ and for users of an advanced tactile aid from the 1990s (Tactaid VII)^28^.

**Table 2:** Consonant and vowel pairs used in the experiment, grouped by the type of contrast.

Consonants	Contrast type	Vowels	Contrast type
/t/&/p/	Place in voiceless plosives	/ɪ/&/ɑː/	Monophthongs
/t/&/k/	Place in voiceless plosives	/iː/&/æ/	Monophthongs
/k/&/p/	Place in voiceless plosives	/ɔː/&/ɪ/	Monophthongs
/f/&/θ/	Place in voiceless fricatives	/ʊ/&/ɑː/	Monophthongs
/f/&/s/	Place in voiceless fricatives	/uː/&/ʌ/	Monophthongs
/ʃ/&/s/	Place in voiceless fricatives	/æ/&/e/	Monophthongs
/d/&/b/	Place in voiced plosives	/ʊ/&/ɪ/	Monophthongs
/g/&/d/	Place in voiced plosives	/æ/&/ɒ/	Monophthongs
/g/&/b/	Place in voiced plosives	/iː/&/uː/	Monophthongs
/v/&/ð/	Place in voiced fricatives	/ʌ/&/æ/	Monophthongs
/v/&/z/	Place in voiced fricatives	/uː/&/ʊ/	Monophthongs
/ð/&/z/	Place in voiced fricatives	/iː/&/e/	Monophthongs
/l/&/r/	Place in sonorants	/ɔɪ/&/eɪ/	Diphthongs
/j/&/l/	Place in sonorants	/ɔɪ/&/aʊ/	Diphthongs
/m/&/n/	Place in sonorants	/aʊ/&/eɪ/	Diphthongs
/z/&/s/	Voicing	/ɪə/&/əʊ/	Diphthongs
/ʒ/&/ʃ/	Voicing	/ʊə/&/eɪ/	Diphthongs
/θ/&/ð/	Voicing	/eə/&/ʊə/	Diphthongs
/t/&/s/	Manner		
/b/&/w/	Manner		
/tʃ/&/ʃ/	Manner		
/ð/&/b/	Manner & place (two-feature)		
/k/&/s/	Manner & place (two-feature)		
/g/&/r/	Manner & place (two-feature)		
/v/&/s/	Place & voicing (two-feature)		
/θ/&/z/	Place & voicing (two-feature)		
/m/&/v/	Place & voicing (two-feature)		

As in previous work^12^, the stimulus duration was matched for all phoneme pairs by fading both stimuli out with a 20-ms raised-cosine ramp, with the exception of pairs containing a diphthong or containing any of the consonants */g/, /d/, /l/, /r/, /v/, /w/,* or */j/*(as production in isolation is impossible or differs acoustically from production in running speech). The ramp reached zero-amplitude at the end of the shortest stimulus (defined as the point at which the signal dropped below 1% of its maximum). This ensured that, for these pairs, discrimination could not be achieved by comparing the durations of the stimuli.

The noise used to mask the phonemes was spectrally shaped to match the long-term average shape of the male and female talker in the EHS Research Group Phoneme Corpus. To establish the long-term average speech spectrum, the short-time Fourier transform (with a window length of 4096 samples) was averaged across all phonemes (with silences removed). A 2049-point FIR filter was then fitted to this spectrum using least-mean squares error minimisation and this was used to filter a 5-min-long white noise.

In each condition containing noise, the noise token was 400 ms longer than the duration of the longest phoneme used in the trial. For each of the three intervals in the discrimination task, the noise token used was extracted from the longer speech-shaped noise sample with a new randomly selected starting point. This meant that each interval contained a different noise token to prevent the participant from identifying the target interval using cues in the noise. The noise was ramped on and off using raised-cosine ramps with a duration of 50 ms. The phoneme onset was delayed from the noise onset by 200 ms. This meant that there was always at least 200 ms between the end of the phoneme and the noise offset.

The SNR was set using the RMS of the phoneme sample after the silences had been removed from the start and end. The start was defined as the point at which the absolute amplitude of the signal first reached 10% of the maximum amplitude of the sample. The end point was defined as the first point from the end of the sample that the absolute amplitude reached 10% of the maximum. The SNR was set to 5 dB (i.e., the RMS of the noise was 5-dB lower than the RMS of the phoneme).

**Figure 4:**
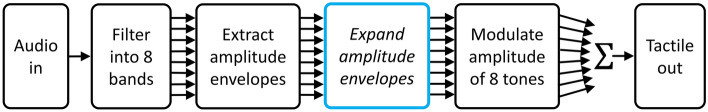
A block diagram showing each processing stage for the tactile vocoder strategy, with the envelope expansion stage highlighted in blue.

The audio-to-tactile conversion was done using a tactile vocoder approach that has been used in previous studies^8–15^, with or without multi-band envelope expansion. Figure 4 shows each processing stage. Before tactile vocoding, the audio intensity was normalised following ITU P.56 method B^57^. The audio was then downsampled to a sampling rate of 16,000 Hz, to match that available in many compact real-time audio devices. Next, the signal was passed through a 512th-order FIR filter bank (stage 2 in Fig. 4). As in Fletcher, et al.^12,15^, eight filters were equally spaced on the auditory equivalent rectangular bandwidth (ERB) scale^58^ between 50 and 7000 Hz. After filtering, the amplitude envelope was extracted for each frequency band using a Hilbert transform and a zero-phase 6th order Butterworth low-pass filter, with a corner frequency of 23 Hz (also matching Fletcher, et al.^12,14,15^). For conditions with expansion, each of the amplitude envelopes was passed through an expansion function similar to that used in previous work^8,11^. Expansion was applied independently to the amplitude envelope from each filter band with an attack and release time of 10 and 100 ms, a slope of 5-dB per octave, and a threshold set to 5-dB below the RMS level of the amplitude envelope. The amplitude envelopes with or without expansion applied (depending on the experimental condition) were then used to modulate the amplitudes of eight fixed-phase vibro-tactile tones.

As in previous work^12,14,15^, the frequencies of the eight tactile tones were 94.5, 116.5, 141.5, 170, 202.5, 239, 280.5 and 327.5 Hz. The frequencies were centred on 170 Hz, which is the frequency at which vibration output is maximal for many compact haptic actuators. The frequencies were spaced based on frequency discrimination thresholds at the dorsal forearm^59^ and remained within the frequency range that can be reproduced by the latest wideband compact haptic actuators. A fixed gain was applied to each vibro-tactile carrier tone to account for differences in sensitivity across frequency, based on tactile detection thresholds^12,42^. The gains were 13.8, 12.1, 9.9, 6.4, 1.6, 0, 1.7, and 4 dB, respectively. The tactile stimuli were scaled so that each had the same RMS amplitude, giving a nominal level of 141.5 dB ref 10^–6^ m/s^2^ (1.2 G). This intensity can be produced by a range of compact, low-powered haptic actuators that could be deployed in a wrist-worn device. The overall amplitude of each stimulus was roved by 3 dB around the nominal level (with a uniform distribution) so that phonemes could not be discriminated using absolute intensity cues. To mask any auditory cues, a pink noise was presented through headphones at 60 dBA.

### Apparatus

During screening and testing, participants sat in a vibration isolated, temperature-controlled room (average temperature of 23 °C, with a standard deviation of 0.45 °C). The room and skin temperature were measured using a Digitron 2022 T type K thermocouple thermometer. The thermometer was calibrated following ISO 80601–2-56:2017^60^ using the method described by Fletcher, et al.^12^.

During screening, vibro-tactile detection threshold measurements were made using a HVLab Vibro-tactile Perception Meter^61^. This has a circular probe with a 6 mm diameter, which contacts the skin through a circular opening in a rigid surround that has a 10 mm diameter. The probe gave a constant upward force of 1N. The downward force applied by the participant was measured using a sensor built into the rigid surround. This was calibrated using Adam Equipment OIML calibration weights and the force applied was displayed to the participant. The output vibration intensity was calibrated using the Vibro-tactile Perception Meter’s built-in accelerometers (Quartz Shear ICP, model number: 353B43) and a Brüel & Kjær (B&K) Type 4294 calibration exciter. All stimuli had a total harmonic distortion of less than 0.1% and the system conformed to ISO-13091–1:2001^62^.

For the phoneme discrimination task, the EHS Research Group haptic stimulation rig was used^12^. This consisted of a Ling Dynamic Systems V101 shaker suspended from an aluminium strut frame by an adjustable elastic cradle. The participant rested their palmar forearm on a foam surface (with a thickness of 95 mm). A 3D-printed (polylactic acid) circular probe with a 10-mm diameter was attached to the shaker, which contacted the dorsal wrist. The probe applied a downward force of 1N, which was calibrated using a B&K UA-0247 spring balance. The shaker was driven using a MOTU UltralLite-mk5 sound card, RME QuadMic II preamplifier, and HV Lab Tactile Vibrometer power amplifier. The vibration output was calibrated using a B&K 4533-B-001 accelerometer and a B&K type 4294 calibration exciter. All stimuli had a total harmonic distortion of less than 0.1%.

Masking noise was played to the participant from the MOTU UltralLite-mk5 sound card through Sennheiser HDA 300 headphones. The audio was calibrated using a B&K G4 sound level meter, with a B&K 4157 occluded ear coupler (Royston, Hertfordshire, UK). The sound level meter calibration was checked using a B&K Type 4231 sound calibrator.

### Procedure

For each participant, the experiment was competed in a single 2-hour-long session. After arriving, participants first gave informed consent to take part and completed a screening questionnaire. This ensured that they (1) did not suffer from conditions that might affect their sense of touch, (2) had not had any injury or surgery on their hands or arms, and (3) had not been exposed to intense or prolonged hand or arm vibration at any time over the previous 24 h. Their self-reported hearing health was also recorded at this stage.

Next, the participant’s skin temperature was measured on the index fingertip of the dominant hand. Participants continued the screening when their skin temperature fell between 27 and 35 °C, as vibro-tactile detection thresholds are known to be effected by skin temperature^63^. At this point, vibro-tactile detection thresholds were measured at the index fingertip following BS ISO 13091–1:2001^62^. During detection threshold measurements, participants applied a downward force of 2N (monitored using the HVLab Vibro-tactile Perception Meter display). Participants were allowed to continue if they had touch perception thresholds in the normal range (< 0.4 m/s^−2^ RMS at 31.5 Hz and < 0.7 m/s^−2^ RMS at 125 Hz), following BS ISO 13091‑2:2021^64^. The fingertip was used for health screening as normative data was not available for the wrist. If participants passed all screening stages, their wrist dimensions were measured at the position they would usually wear a wristwatch and they continued to the experiment phase.

In the experiment phase, the skin temperature was measured on the dorsal wrist at the position where the participant would normally wear a wristwatch (the site at which tactile stimulation was delivered during the experiment). The skin temperature at the wrist was required to be between 27 and 35 °C. Participants then sat in front of the EHS Research Group haptic stimulation rig, with the probe from the shaker contacting the dorsal wrist. They performed a three-interval, three-alternative forced-choice phoneme discrimination task that was developed previously^12^. In each trial, a single phoneme pair from the male or female talker was used (see “Stimulus”). Two of the three intervals contained one randomly selected phoneme from the pair and the other interval contained the other phoneme. For each phoneme, one of four tokens available for each talker in the corpus was selected at random on each trial. In conditions that included background noise, all three intervals contained noise. Intervals were separated by a gap of 250 ms and the interval order was randomised. The participant’s task was to select via a key press which of the three intervals contained the phoneme that was presented only once. In addition to being instructed about how to perform the task, participants were told to ignore the overall intensity of the vibration in each interval (as level roving was deployed to prevent the use of overall intensity for discrimination). Visual feedback, which indicated whether the response was correct or incorrect, was displayed for 500 ms after each trial.

The percentage of phonemes correctly discriminated was measured across four conditions: (1) without background noise and without envelope expansion, (2) without background noise and with envelope expansion, (3) with background noise and without envelope expansion, and (4) with background noise and with envelope expansion. For each condition, all phoneme pairs (see “Table 2”) were tested twice for both the male and female talker. All phoneme pairs and conditions were measured for each repeat in sequence, with the order of trials randomised each time. There were 720 trials in total for each participant. For each condition there were 180 trials, consisting of 108 consonant contrasts and 72 vowel contrasts. For the subgroup contrasts (Fig. 2.), there 12 trials for each consonant subgroup per participant (3 phoneme pairs, 2 talkers, and 2 repeats), for the vowel monophthong subgroup there were 48 trials, and for the vowel diphthong subgroup there were 24 trials.

The experimental protocol was approved by the University of Southampton Faculty of Engineering and Physical Sciences Ethics Committee (ERGO ID: 68477). All research was performed in accordance with the relevant guidelines and regulations.

### Statistics

The percentage of phonemes correctly identified was calculated for each condition for the male and female talker. Primary analysis consisted of two three-way repeated-measures analyses of variance (RM-ANOVA), with factors ‘Envelope expansion’ (on or off), ‘Phoneme type’ (consonant or vowel), and ‘Talker’ (male or female). The first RM-ANOVA was run on the conditions with no background noise and the second on the conditions with background noise. For both RM-ANOVAs, data were determined to be normally distributed based on visual inspection and on Kolmogorov–Smirnov and Shapiro–Wilk tests. The RM-ANOVAs used an alpha level of 0.05.

Post-hoc analyses were then conducted. The first set of analyses involved four two-tailed *t*-tests assessing whether there was a significant change in performance due to envelope expansion for consonants and vowels, either in quiet or in noise. The second set involved two-tailed *t*-tests assessing the effect of envelope expansion for each phoneme contrast type (see “Table 2”), in quiet and in noise. All tests were had a Bonferroni-Holm correction^65^ for multiple comparisons applied (26 comparisons).

Finally, four Pearson’s correlations were run between either participant age or detection thresholds for a 125-Hz vibro-tactile tone (measured during screening) and overall phoneme discrimination scores in quiet, either with or without envelope expansion. These exploratory additional analyses were not corrected for multiple comparisons as it was expected that no correlation would be found in any of these conditions, following results from previous studies (e.g.,^12,14^).

## Data Availability

The datasets generated and analysed during the current study are available in the University of Southampton’s Research Data Management Repository at: 10.5258/SOTON/D3139.
